# Oscillatory bursting of gel fuel droplets in a reacting environment

**DOI:** 10.1038/s41598-017-03221-x

**Published:** 2017-06-12

**Authors:** Ankur Miglani, Purushothaman Nandagopalan, Jerin John, Seung Wook Baek

**Affiliations:** Department of Aerospace Engineering, School of Mechanical and Aerospace Engineering, Korea Advanced Institute of Science and Technology (KAIST), Daejeon, 34141 Republic of Korea

## Abstract

Understanding the combustion behavior of gel fuel droplets is pivotal for enhancing burn rates, lowering ignition delay and improving the operational performance of next-generation propulsion systems. Vapor jetting in burning gel fuel droplets is a crucial process that enables an effective transport (convectively) of unreacted fuel from the droplet domain to the flame zone and accelerates the gas-phase mixing process. Here, first we show that the combusting ethanol gel droplets (organic gellant laden) exhibit a new oscillatory jetting mode due to aperiodic bursting of the droplet shell. Second, we show how the initial gellant loading rate (GLR) leads to a distinct shell formation which self-tunes temporally to burst the droplet at different frequencies. Particularly, a weak-flexible shell is formed at low GLR that undergoes successive rupture cascades occurring in same region of the droplet. This region weakens due to repeated ruptures and causes droplet bursting at progressively higher frequencies. Contrarily, high GLRs facilitate a strong-rigid shell formation where consecutive cascades occur at scattered locations across the droplet surface. This leads to droplet bursting at random frequencies. This method of modulating jetting frequency would enable an effective control of droplet trajectory and local fuel-oxidizer ratio in any gel-spray based energy formulation.

## Introduction

The success of future rocket propulsion systems will depend on their ability to utilize eco-friendly fuels that exhibit high exothemicity, shortened ignition delay time, high energy density and operational safety at low cost (i.e. easy to store and handle)^1, 2^. In this light, there has been a considerable interest in high-performance gellant based fuels as alternatives to conventional neat propellants^3–10^. This is due to their enhanced rheophysical properties that syndicate the advantages of both solid and liquid fuel propellants. For instance, a solid-like behavior at low or negligible shear reduces the chance of a hazard due to accidental spill or leakage (particularly in case of toxic and hypergolic propellants), thus, facilitating a safe handling and storage^11^. High viscosity during storage also enables a stable and homogenous suspension of energetic nanoscale additives in the fuel matrix which increases the fuel energy density and specific impulse of the engine. On the other hand, liquid-like behavior at high shear offers the flexibility of thrust modulation and re-ignitability in engines. Furthermore, the shear thinning property ensures that the gelled fuels can be pumped easily into the combustion chamber like liquid propellants and aid an effective atomization when forced through injectors^12–17^.

In spite of these potential advantages, gel fuels exhibit complex combustion and rheological characteristics compared to solid/liquid propellants. For instance, disruptive burning of gel fuels is highlighted by jetting of unreacted fuel vapors^18^ that occurs asymmetrically and randomly during its lifetime. A typical jetting event involves four key stages that occur sequentially and at spatially distinct locations^19^. First, gellant crust/shell formation due to phase seperation of gelling agent from the base fuel/gellant solvent (near the droplet surface). Second, boiling of trapped fuel (inside the droplet). Third, bursting of the gellant shell due to internal pressure build-up and jetting of unreacted fuel vapors (across the droplet-gas interface) and fourth, jet travel to the flame envelope: which causes distortion in the symmetric tear-drop shaped flame geometry.

It is noteworthy to consider that the terms *jetting* and *microexplosion* have been used interchangeably in the existing literature for highlighting disruptive burning of gelled fuel droplets. However, they represent a completely different class of disruption events. As first reported by Law^20–22^, microexplosion corresponds to a sudden catastrophic break-up of the droplet that occurs due to rapid internal gasification of high volatility component. Specifically, the low boiling point species is diffusionally entrapped in the droplet core and gets superheated to its homogenous boiling limit, thereby, resulting in an immense pressure build-up and catastrophic breakup of the droplet. This occurs only once and marks an end to the droplet lifetime. Unlike microexplosion, jetting events occur throughout the droplet lifetime as bubbles form, grow and collapse continuously to expel out the unreacted fuel vapor. Such continuous bubble formation and existence of a large bubble population is typical of heterogeneous boiling. Since heterogeneous boiling features a low degree of stored superheat the jetting events have a noticeably low intensity as opposed to a microexplosion.

With context to heterogeneous boiling, *vapor jetting* to a certain degree is similar to the *bubble ejections* observed during burning of colloidal fuel droplets^23–27^ since latter also features multiple bubble formation. However, in terms of the thermo-physical processes governing their initiation, growth, termination and their post-termination effects on the combustion behavior, vapor jetting and bubble ejections are markedly different disruption modes. Firstly, in case of nanofluid fuel droplets the nanoparticles (NPs) agglomerate to form micro scale aggregates which then act as nucleation sites^23^. In contrast, in gel fuel droplets it is the inner surface of viscoelastic gellant shell that serves as a nucleation surface. Secondly, in nanofuel droplets the internal accumulated pressure is released through rupture of the crust that is formed by the consolidation of nanoparticles at the receding droplet-gas interface. This rupture accompanies the formation of daughter droplets (DDs) that pinch-off from the parent droplet^23, 24^ and may further undergo puffing and microexplosion^28–30^. Contrarily, in gel fuel droplets the rupture of gellant shell is not accompanied by the formation of any DDs but results in jetting out of unreacted fuel vapors through ruptured portion of the shell, thereby releasing the internal pressure. Thirdly, in nanofuel droplets the shell formation process is governed by coupled but competing mechanisms of NP agglomeration and secondary atomization that alter the mass fraction of NPs within the droplet dynamically. The former aids shell development through particle aggregation while the latter tends to disrupt the shell directly through rupture and indirectly by continuous efflux of NPs^26^. However, in gel fuel droplets the shell formation occurs through phase separation of the gellant^18, 19, 31–35^ from the base fuel/gellant solvent/gellant formulation and the whole gellant mass is retained within the droplet domain until it carbonizes and gets consumed towards the end of droplet lifetime. Finally, in nanofuels and in general in other multi-phase multicomponent droplets, the formation of DD’s (secondary atomization) and their subsequent break-up (tertiary atomization)^29^ facilitates an enhancement in burn rate by increasing the net surface area and distributing the fuel charge uniformly. Additionally, the DD’s act as carriers through which the energetic nanoparticles present within the droplet domain are transported to the flame to harness their energy^25, 27^. However, the significance of jetting observed in case of gel fuel droplets lies in its ability to carry the unreacted fuel from the droplet to its surrounding. This enhances the fuel mass flux, thereby, facilitating faster burn rates. This is particularly crucial for rocket motor applications utilizing gel propellants since gels exhibit a higher ignition delay and low burning rates due to their inherently high heat of vaporization^36^. By utilizing high-speed OH-PLIF in burning monomethyhyrazine (MMH) droplets (gelled with Hydroxypropyl cellulose HPC), Cho *et al*.^37^ illustrated that such disruptive burning exhibits a tri-modal behavior. First, the jets may corrugate the flame envelope. Second, the jets may break flame envelope (i.e. localized extinction) and, third, jets form a local fireball outside the flame envelope. The occurrence of a particular disruptive mode depends on the jetting intensity (i.e. maximum jet speed) which is highest for mode 3 (~0.8 m/s) and lowest for mode 1 (~0.35 m/s). Previous studies have shown that the jetting velocity can be altered by varying initial droplet diameter^38^, regulating external ambient conditions^33, 37, 38^ and varying the functional properties such as the parent fuel, initial gellant concentration/type (organic or inorganic gellants)^19, 34, 37, 39^ and through addition of energetic nanophase additives (metallized gels). Literature search reveals that most studies have focused on jet visualization at the flame scale while the identification of different jetting modes, their initiation mechanisms and methods to control them at the droplet scale has received little attention.

From a viewpoint of combustion efficiency organic gellants offer several advantages compared their inorganic counterparts: First, unlike inorganic gellants that are inert the organic gellants are consumed completely during the burning process and hence they contribute to the heat of combustion with no left over residue. Secondly, inorganic gellants aid in the formation of a rigid, impervious shell which eventually leads to droplet microexplosion, thereby, displaying an uncontrolled atomization. In contrast, organic gellants form a viscoelastic shell that ruptures and re-establishes as jetting events proceed. This offers an opportunity of modulating the jetting characteristics (intensity and frequency) by tuning the shell properties simply by varying the gellant concentration. This is particularly attractive since it offers an ease of control at the initial stage of fuel formulation. We exploit this behavior to show that: (1) an eco-friendly ethanol gel droplet containing a cellulose based derivative as the gelling agent (Hydroxy methyl Propyl Cellulose: HPMC organic gellant) displays *oscillatory jetting* in a reacting environment due to acyclic bursting of its shell. (2) By varying the initial gellant loading (3 to 6 wt.%) the bursting frequency can be modulated and (3) by applying set-theory to the rupture jet holes (in the shell) different jetting states of the droplet can be identified i.e. coincidental, off-set or displaced.

A fundamental understanding of oscillatory droplet combustion is also crucial from an application viewpoint given its relevance to combustion instabilities^40–43^ in liquid rocket engines. Interestingly, previous studies have shown that the pure fuel (ethanol, methanol) droplet flames are also susceptible to oscillations when subjected to external acoustic excitation either through travelling^24, 44^ or standing pressure waves^40–43^. In the latter case, an increase in droplet burn rate by as large as ~15% under normal gravity conditions has been reported when the sound pressure level exceeded ~135 dB and droplet was placed near pressure node. On the contrary, there was a little observable change in gasification rate when droplet was stationed at velocity node. Further, the droplet diffusion flames were found to be most responsive at low excitation frequencies (~5–330 Hz)^24, 40–44^ while they exhibited diminished response at higher forcing frequencies (>400 Hz). This indicates that oscillation mechanisms in combusting droplet exhibit multi-parameter dependence and hence provide an innovative means of manipulation at the droplet scale, which form a sub-grid elements of any gel spray based combustion strategy.

## Results and Discussion

**Figure 1:**
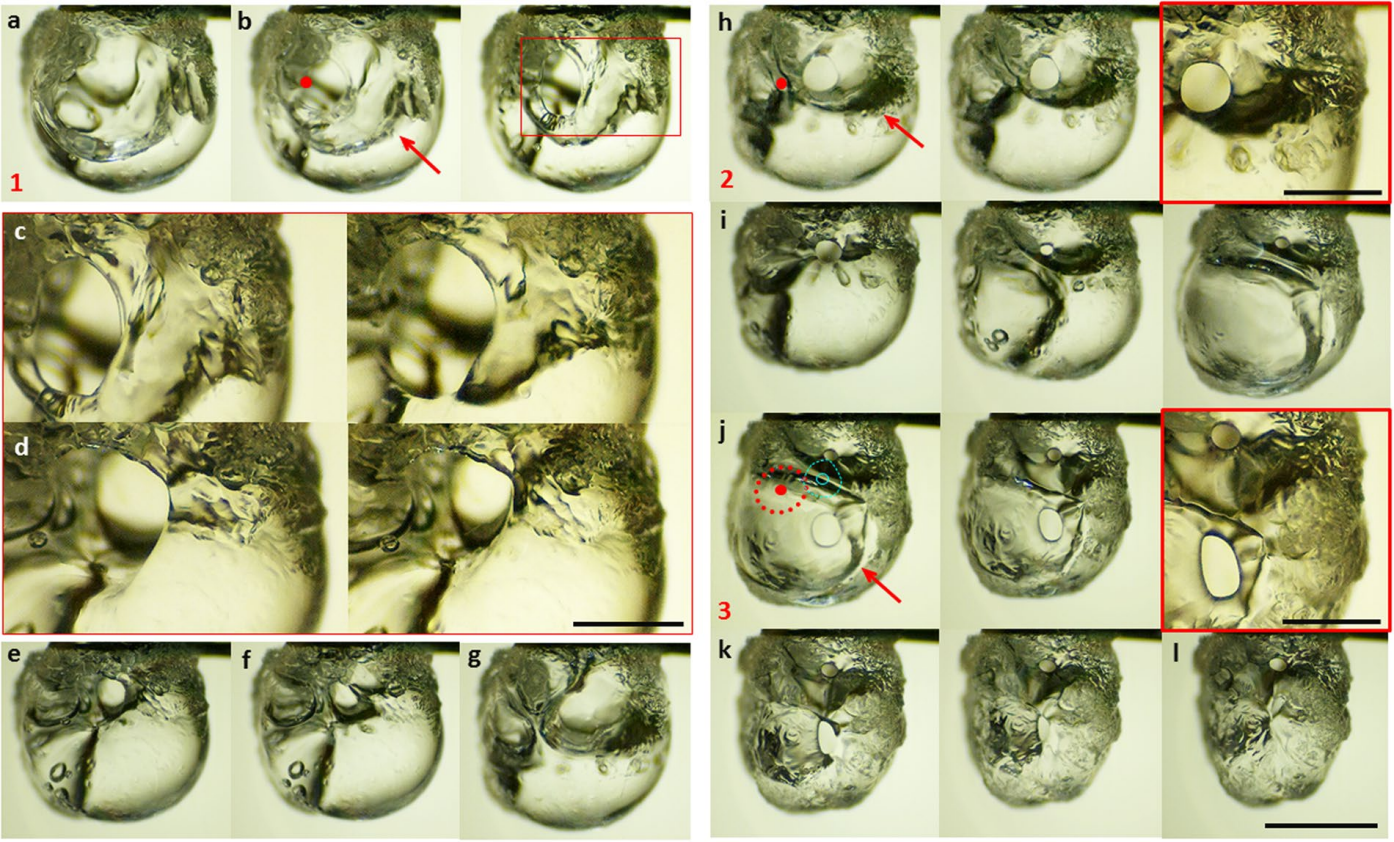
Dynamic rupture sequence of a burning organic gellant (3 wt.% HPMC) based ethanol fuel droplet undergoing an oscillatory jetting cascade with three cycles. Cycle 1: (**a**) Swelled droplet state; *t* = 0 ms, (**b**) First gellant layer rupture leading to jet hole formation; 0.834 and 1.527 ms. Red dot indicates the initial rupture location. Red arrow marks the feeder bubble that acts as a source of unreacted fuel vapor during jetting. (**c**) Magnified view showing jet hole expansion following initial rupture; 1.945 and 4.027 ms, (**d**) magnified view of jet hole during retraction; 8.612 and 12.361 ms, (**e**) continued retraction; 15.416 ms, (**f**) complete recovery of ruptured site; 86.667 ms and (**g**) bubble growth leading to pressure build-up; 88.194 ms. (**h**) Second rupture leading to droplet deflation and marking onset of Cycle 2; 88.75, 89.305 and 89.723 ms. (**i**) Continual shrinkage and displacement of the active jetting site alongside pressure build-up through bubble growth in the bottom hemisphere; 92.916, 131.112 and 388.055 ms. (**j**) Third rupture leading to simultaneous jetting through two active sites (Cycle 3); 388.75, 389.723 and 391.527 ms. *Weak soft region* of the droplet surface is shown superimposed. (**k**) Continuous shrinkage of bottom jetting site alongside sustained jetting from top jetting hole; 393.612 and 397.778 ms. (**l**) complete recovery of bottom site but sustained jetting from top; 403.334 ms. Images (**a**) to (**f**) and (**h**) to (**l**) constitute the active jetting ${t}_{a}$ period while (**f**) to (**g**) form the inactive or pressure build-up period ${t}_{ia}$. The scale bars equal 1 mm and 0.5 mm for images with overall view and magnified view respectively.

Figure 1 illustrates the dynamic bursting sequence of a gelled ethanol droplet undergoing an *oscillatory rupture cascade*. Such cascades occur intermittently during the intermediate stages of droplet lifecycle and each cascade constitutes a series of *rupture cycles* that occur aperiodically. Further, as shown in Fig. 1, each cycle features a sequential *four stage* process of: (1) Gellant layer rupture at a weak soft spot. This is a key stage that results in the hole formation and provides an opening for the outflux of unreacted fuel vapors via jetting. (2) Continuous jetting alongside expansion of the rupture site. (3) Continuous jetting alongside shrinkage of the ruptured site; eventually leading to the recovery of ruptured layer and (4) bubble growth period, which features an internal pressure build-up and initiation of next rupture cycle. Clearly, the last stage corresponds to a dormant or *inactive period* (${t}_{ia}$) where the internal pressure rises while the first three stages are associated with pressure release through *active jetting* (${t}_{a}$). Thus, an oscillatory cascade forces the droplet into irregular inflation–deflation cycles coupled with the alternate jetting. As these jets hit the flame envelope repeatedly, they induce flame envelope oscillations that are synchronized but out-of-phase with bulk droplet shape oscillations (droplet shrinkage corresponds to active jetting and vice versa). Supplementary Videos 1 and 2 illustrate how the droplet and its surrounding flame envelope behave as an in-sync driver-driven system, where alternative jetting is imposed on the flame and leads to oscillatory disruptive burning. Oscillatory flame response is described in Supplementary information.

**Figure 2:**
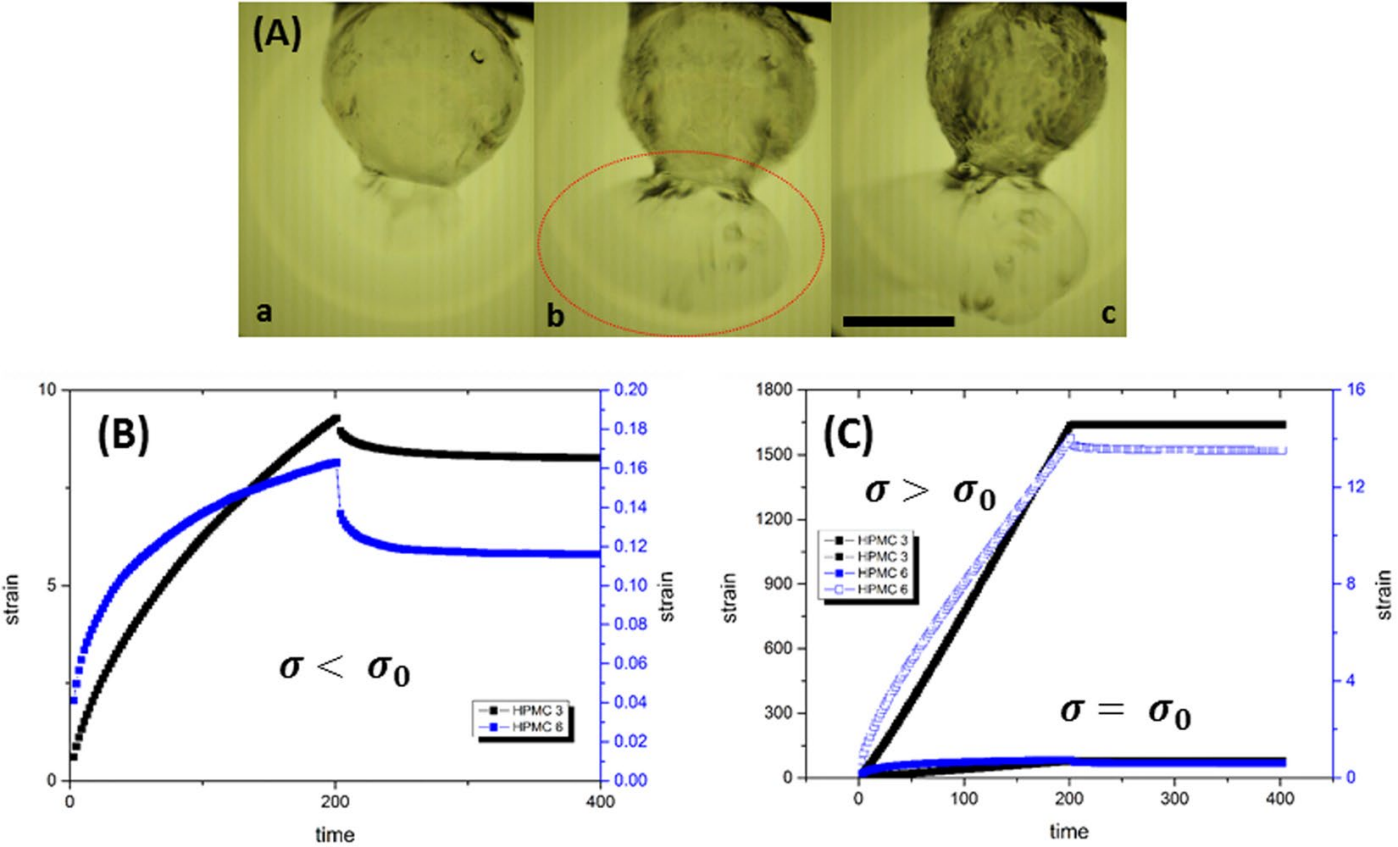
(**A**) Sequential process of elongation, thinning and yielding of the shell prior to rupture. (a) Bulging out of the shell from a weak sector causing stretching; *t* = 0 ms (b) *ballooning* of the bulged portion leading to severe stretching and possible yielding; thin sheet formation; 0.138 ms. (c) sheet rupture; 0.277 ms. Image sequence is for a burning ethanol gel droplet with 3 wt.% gellant loading. The scale bar equals 1 mm. Temporal variation of creep-strain as a function gellant loading rate for HPMC 3 and HPMC 6 at different shear ($\sigma$) loading conditions: (**B**) below yield point ($\sigma =10\,{\rm{Pa}} < {\sigma }_{0}$), (**C**) at yield point and above yield point.

During a cascade, the tendency of droplet bursting at any given time instant is governed by a direct competition between the ebullition activity that tends to scale-up internal pressure and the ability of viscoelastic shell to sustain this pressure upsurge. Before the shell ruptures it restrains pressure rise through stretching, thinning down and yielding as shown in Fig. 2A. Therefore, the pressure build-up pathway leading to cascades is a strong function of rheophysical properties of the crust such as the yield stress $({\sigma }_{0})$, strain (*γ*: before and after yielding) and the thickness (*δ*) that vary with GLR. A physical analysis of the shell bursting dynamics is discussed in Supplementary information. Note that it is extremely difficult to predict the spatio-temporal variation of shell properties in a reacting environment where frequent ruptures are altering the shell dynamically. In this light, the rheometric measurements of gel fuel properties under isothermal conditions can provide critical insights on the oscillatory bursting behavior of droplets. For instance, Table 1 shows that the yield stress (obtained from simple shear flow study) increases monotonically with the GLR. Specifically, $\frac{{\sigma }_{0,6wt. \% }}{{\sigma }_{0,3wt. \% }}\cong 5.2$ and $\frac{{\sigma }_{0,4or5wt. \% }}{{\sigma }_{0,3wt. \% }}\cong 2$ are indicative that a high gellant loading facilitates a much stronger crust formation. However, the dynamic creep analysis (Fig. 2B and C) that predicts the ability to undergo deformation indicates that for the same magnitude of applied shear stress (below ${\sigma }_{0}$) the 3 wt.% gel fuel undergoes strain that is an order higher compared to 6 wt.%. Furthermore, at and beyond their respective yield points, 3 wt.% endures even larger strains i.e. $\frac{{\gamma }_{3wt. \% }}{{\gamma }_{6wt. \% }} \sim \,O({10}^{2})$. Thus, on a comparative basis, a high GLR (4, 5 and 6 wt.%) aids in a *strong* but *rigid* shell formation i.e. high ${\sigma }_{0}$ but small $\gamma$, while a low GLR (3 wt.%) results in a *weak* but *flexible* shell formation i.e. low ${\sigma }_{0}$ but large $\gamma$. For brevity, subsequent discussion is done based on representative cases i.e. 3 wt.% for low GLR and 5 wt.% for high GLR.

**Table 1:** Relative composition (in weight %) and yield stress of test fuels.

Fuel Sample	Research Grade Ethanol (C_2_H_5_OH)	De-ionized Water (H_2_O)	Hydroxypropyl Methyl Cellulose	Yield Stress ${{\boldsymbol{\sigma }}}_{{\bf{0}}}({\boldsymbol{Pa}})$
HPMC 3	82	15	3	23.23 ± 2.62
HPMC 4	81	15	4	48.94 ± 4.55
HPMC 5	85	10	5	50.46 ± 4.52
HPMC 6	84	10	6	120.58 ± 5.22

**Figure 3:**
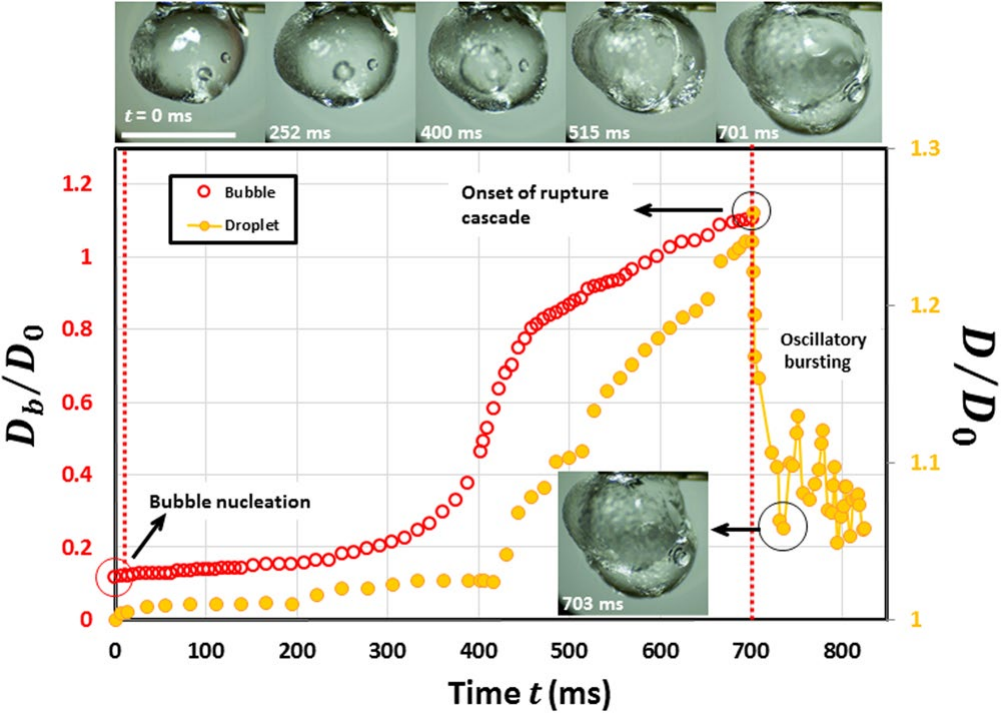
Temporal variation of non-dimensional bubble $({D}_{b}/{D}_{0})$ and droplet diameter $(D/{D}_{0})$ at pre-cursor stages of first oscillatory rupture cascade in a combusting ethanol gel droplet (${D}_{0}\, \sim$ 1.72 mm). The scale bar in droplet image equals 2 mm.

**Figure 4:**
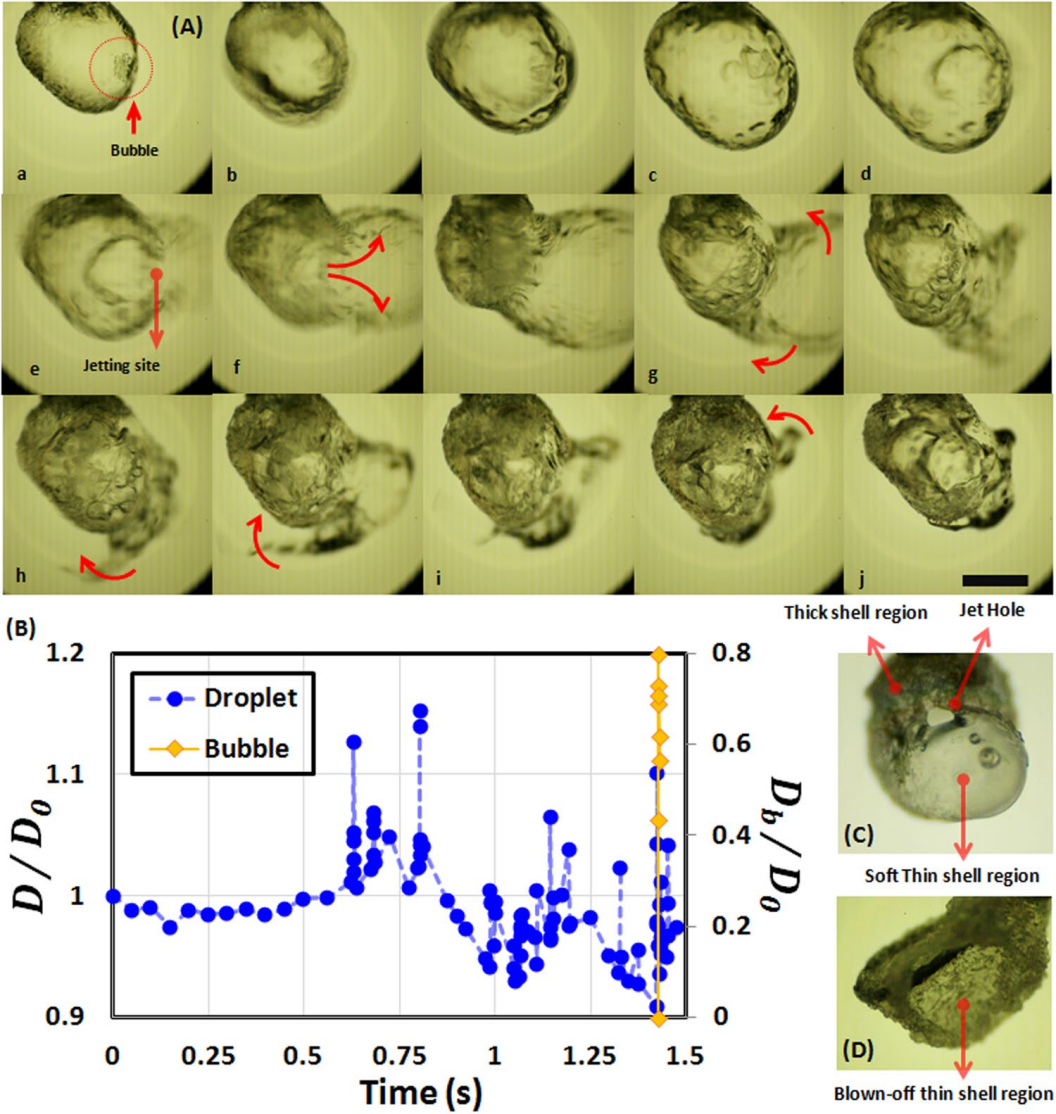
Bursting dynamics of a 5 wt.% gelled ethanol fuel droplet undergoing combustion. (**A**) High intensity rupture featuring a sequential transition from an initial smooth shell to a final inhomogeneous deformed state: (a) bubble nucleation; *t* = 0 ms, (b) rapid bubble expansion and coupled droplet swelling; 0.277, 0.416 ms, (c) state of maximum droplet dilatation; 0.833 ms, (d) Onset of rupture at weak soft spot; 0.972 ms, (e) shell collapse and formation of jetting site; 1.112 ms, (f) continual expansion of ruptured layer/ sheet due to rupture impact; 1.25, 1.527 ms, (g) sheet retraction; 1.945, 2.223 ms, (h) sheet collapse back onto the droplet surface at random locations; 2.638, 3.194, 3.472, 3.889 ms and (j) formation of a new non-uniform shell; 5 ms. The scale bar equals 1 mm. (**B**) Time history of normalized bubble $({D}_{b}/{D}_{0})$ and droplet diameter $(D/{D}_{0})$. Transient spikes correspond to pressure surges resulting from rapid bubble growth. (**C**) Microscopic image of the final combustion residue showing an inhomogeneous shell structure and (**D**) Weak, thin portion of the shell is blown-off during a high intensity rupture leaving behind a hollow shell.

Since shell constitutes outermost layer of the droplet, the formation of distinct shells with variation in GLR is reflected directly in the droplets response to internal pressure rise during the precursor stages leading to first cascade. Figures 3 and 4 show a comparison of the precursor bubble growth event and the coupled droplet response as a function of GLR. Clearly, at low concentrations (Fig. 3) gel fuel droplet features a slow and continuous bubble growth (${\dot{R}}_{b}$ ~ *O* (1.5 mm/s); ${R}_{b}\,$is the bubble diameter) which extends over large timescales *O*(1000 ms) i.e. comparable to the droplet lifetime. Besides, just prior to rupture the bubble grows to a markedly high droplet void fraction (~0.7) which causes severe droplet swelling (${D}^{3}/{D}_{0}^{3} \sim 2)$. A large volumetric dilatation at low GLR suggests that the droplet accommodates internal pressure rise dynamically through continuous expansion and stretching of the shell. This inturn leads to a gradual pressure build-up on the way to cascade onset.

**Figure 5:**
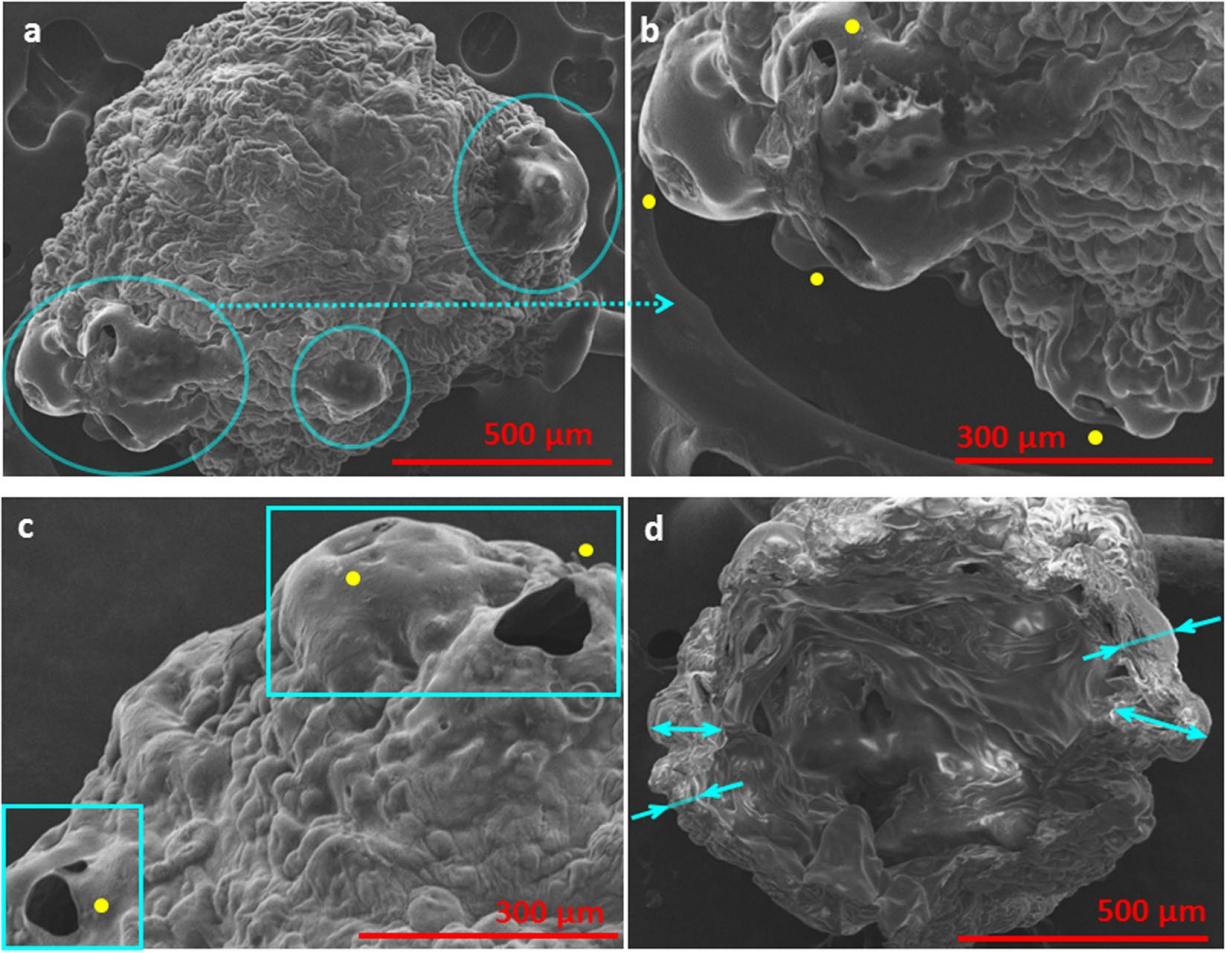
SEM micrographs showing surface morphology of the final combustion residue for a 5 wt.% gelled ethanol fuel droplet: (**a**) Overall view showing a deformed shell structure; blue circles indicate non-uniformly distributed soft regions (preferred rupture sites) with jetting holes, (**b**) magnified view of a soft region showing location of jetting holes (yellow dot markers), (**c**) magnified image demarcating rigid and soft regions of the shell and (**d**) Overall cross-sectional view of final hollow microstructure with non-homogenous shell thickness. This also delineates the smooth inner surface with the rigid outer skin. Magnification for Fig. a–d equals 80X, 150X, 180X and 90X respectively.

In contrast, at 5 wt.% (Fig. 4), bubble lifecycle is characterized by transient periods of rapid expansion and contraction that occurs over very short timescales ~*O* (5 ms) (seen as transient spikes in time history of droplet size: *D*/*D*_0_; Fig. 4B). This is because as the bubbles tend to grow, a strong-rigid shell inhibits droplet expansion which inturn leads to pressure accumlation within the droplet domain, thereby, forcing the bubble to contract back. In this regard, droplets with high GLR are in an unstable state as they are continuously subjected to transient pressure surges. However, as the droplet lifetime proceeds and bubbles receive continuous heat flux from the surrounding flame envelope they finally expand profusely tearing apart the shell, as shown in Fig. 4A(a–e). Following such high intensity rupture the shell is completely rearranged and leads to a new deformed droplet shape (Fig. 4A,j). Figure 4A(g–i) illustrates that as the ruptured layer retracts it settles randomly on the droplet surface, thereby, inducing a thickness inhomogeneity in the shell structure. Such collapsing back of the layer (following rupture) onto the droplet surface to a form a new shell has also been reported previously^35^. It is important to note here that starting from an initial GLR the shell is first formed as the high volatility component (i.e. ethanol) is preferentially vaporized and the gellant concentration at the droplet surface reaches a critical concentration (~0.9–0.95)^28, 35^. This signifies that the droplet core would always be at a lower gellant concentration compared to the shell^28, 35^. Therefore, following a high intensity rupture, the shell gets thinner in the ruptured region as this region is exposed to fresh gel from the droplet core while it gets thicker in the surrounding regions where the retracting sheet overlaps with the previously intact layer. In this new state, the ruptured region becomes a weak soft part in an otherwise strong crust and acts as a favored site for cascades. Formation of a weak region alongside jetting hole is shown in Fig. 4C and D. This is also evident from the SEM micrographs of the combustion residue in Fig. 5 (for 5 wt.% case) which shows the presence of jetting holes in soft regions while corrugated thick regions are intact.

Additionally, SEM image of cut-section through the final precipitate (Fig. 5d) illustrates the shell thickness inhomogeneity and a highly deformed droplet shape resulting from several high intensity bursting events that occurred during its lifetime. This is contrary to the findings of previous studies^28, 35^ where a uniform shell formation was reported during combustion of gel fuel droplets. The jet hole depth is used to estimate the shell thickness *δ* averaged over multiple points along the circumference and found to be ~30 ± 15 µm and ~75 ± 20 µm at 3 wt.% and 5 wt.% GLR respectively. Also, average *δ* determined from crossectional view of the hollow precipitate for 5 wt.% case (Fig. 5d SEM image) is found to be of the same order ~*O* (110 µm). Thus a higher GLR leads to a thicker shell on an average.

Furthermore, since high intensity bursting is driven by transient pressure spikes it tends to occur randomly across the droplet surface. This creates an entirely distinct and new shell configuration with each rupture. Given the complexity of shell rupture-reformation process at 5 wt.%, the probablity of randomly distributed oscillatory jetting sites across the droplet surface is quite high. Indeed, SEM images of Fig. 5 show that weak sites that act as preferred locations for onset for cascades are scattered non-uniformly. This inturn causes successive cascades to occur at random spatial locations at high GLR. In summary, at low GLR, first cascade is triggered through a single step, gradual pressure build-up process due to the presence of thin-weak-flexible shell while at high GLRs it initiates through intermittent pressure surges (i.e. the crust is debilitated in steps) due to a thick-strong-rigid shell. It is important to note that although the pressure build-up pathways leading to a cascades vary markedly with the GLR, the actual cascades proceed as shown in Fig. 1 and is generic for all cases.

### Effect of gellant loading on global bursting dynamics

**Figure 6:**
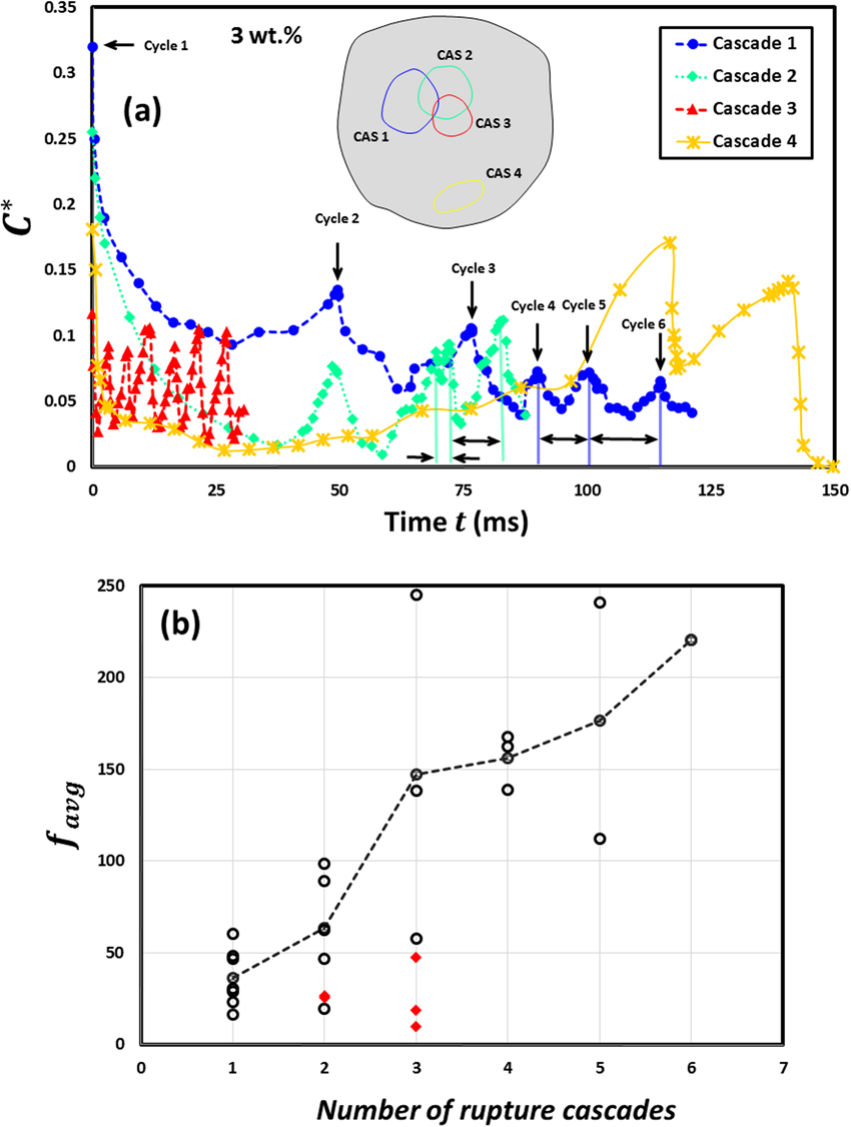
Time evolution of global oscillatory response parameters for a 3 wt.% gelled ethanol fuel droplet undergoing burning. (**a**) Variation of circumferential strain $({C}^{\ast }=\Delta C/C)$ across different oscillatory rupture cascades. $C=2\sqrt{\pi {A}_{p}}$ (${A}_{p}\,$is projected droplet area) is the reference circumference in the deflated droplet state prior to onset of a rupture cascade. (**b**) Average bursting frequency ${f}_{avg}=$ $\frac{1}{{\sum }_{N}({t}_{a}+{t}_{ia})/N}$ of the droplet as a function of cascade count.

Temporal fluctuations in circumferential strain (${C}^{\ast }=\Delta C/C$) in Fig. 6 illustrate the oscillatory response of a 3 wt.% ethanol gel droplet undergoing combustion. Here, $C$ is the reference circumference that is specific to each cascade and calculated at a precursor stage when the droplet just begins to dilate on the way to first rupture cycle. This time varying term accounts for the change in droplet circumference due to a reduction in its size as the solvent mass gets depleted through vaporization and jetting. From a global perspective at the droplet scale, Fig. 6a for 3 wt.% case indicates that at onset of first cascade (at a given location), the shell is stretched substantially due to droplet expansion (${C}_{max}^{\ast }$ > 0.3) which causes it to yield and thin down. As a result, the maximum circumferential strain ${C}_{max}^{\ast }$ the droplet can sustain in ensuing cycles (before shell ruptures) decays drastically and reduces by ~80% over a span of 6 cycles. This also indicates that due to repeated ruptures (or increasing number of cycles) this region of the droplet becomes progressively weaker. At this stage (i.e. following the end of first cascade), subsequent cascades may either occur in this weak region or at a different location on the droplet surface. Inset in Fig. 6a depicts these potential states schematically, where cascades CAS 1, 2 and 3 are confined to same region while CAS 4 occurs separately at a distinct location. Henceforth in the text the cascades will be denoted as CAS *i* while the rupture cycles will be denoted by CAS *i* C *j*, where *i* and *j* = 1, 2, 3… represent the indices for ${j}^{th}$ cycle in a ${i}^{th}\,\,$cascade. In the former case, as the gellant layer is already weakened by CAS 1, ${C}_{max}^{\ast }$ at first cycle decays monotonically during ensuing cascades while it is significantly small (${C}_{max}^{\ast }\, \sim \,\,$0.1) for remaining cycles. Also, as the shell gets more and more weak with successive cascades it is prone to frequent ruptures. This is evidenced by Fig. 6b which shows an increase in the average bursting frequency ${f}_{avg}$ $=\frac{1}{{T}_{avg}}=\frac{1}{{\sum }_{N}({t}_{a}+{t}_{ia})/N}$ (where *N* is total number of cycles in a given cascade) by an order of magnitude i.e. from ${f}_{avg}\cong$ 44 Hz in CAS 1 to 228 Hz in CAS 6. Contrarily, the latter case of CAS 4 occurring at a spatially distinct location features a higher ${C}_{max}^{\ast }$. Figure 6b demarcates these two cases based on ${f}_{avg}\,$as a function of cascade count. Clearly, ${f}_{avg}$ increases continuously when all six cascades occur in same region of the droplet while it reduces for isolated cascades (CAS 2 and CAS 3 occurring at distinct locations have lower bursting frequency ${f}_{avg}\cong$ 14 Hz). This may be explained as follows: CAS 4 occurs at a location which has not undergone any prior cascade, therefore, while the fuel mass is being depleted through jetting via CAS 1, 2 and 3 and vaporization the gellant would accumulate in regions not affected by these cascades. The shell is then expected to become thicker in these regions, thereby requiring a larger strain to rupture. The very occurrence of distributed cascades also explains why a non-homogenous shell is formed in combusting gel fuel droplets even at low GLR.

**Figure 7:**
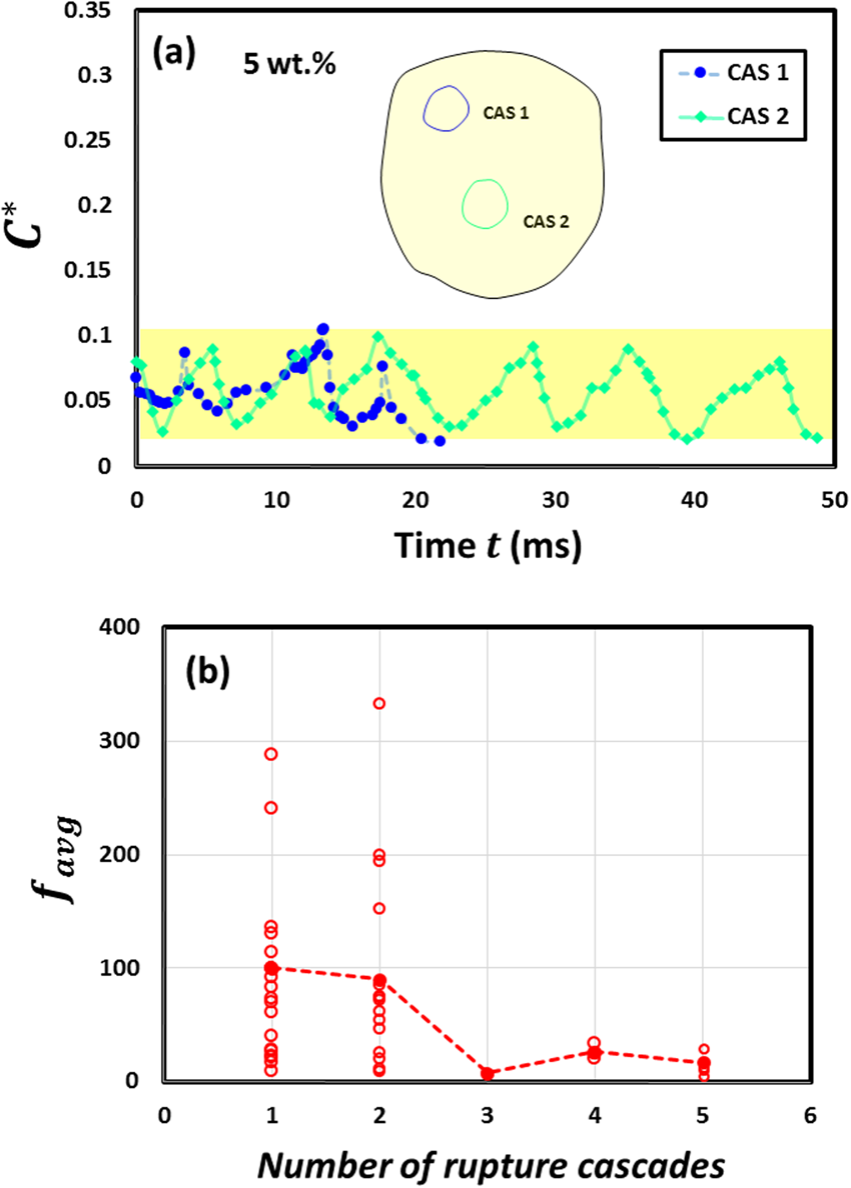
Time evolution of global oscillatory response parameters for a 5 wt.% gelled ethanol fuel droplet undergoing burning. (**a**) Variation of circumferential strain $({C}^{\ast }=\Delta C/C)$ across different oscillatory rupture cascades. (**b**) Average bursting frequency ${f}_{avg}=$ $\frac{1}{{\sum }_{N}({t}_{a}+{t}_{ia})/N}$ of the droplet as a function of cascade count.

However, for 5 wt.%, Fig. 7a shows that ${C}_{max}^{\ast }$ at rupture is significantly low and confined to a narrow band (${C}_{max}^{\ast }$ varies from ~0.06–0.1) for all cycles and across all cascades. This reveals two key findings: First, since ${C}_{max}^{\ast }$ is a global parameter, a small variation in ${C}_{max}^{\ast }$ means that high GLRs result in a stronger-rigid crust (compared to low GLRs) that undergoes minimal strain as a whole. Secondly, as ${C}_{max}^{\ast }$ at first cycle does not decay across cascades, it reinforces the point that successive cascades do not occur in the same region of the droplet but are distributed rather randomly. This is in line with the discussion from previous section (with reference to Fig. 5 SEM) and also evidenced by Fig. 7b which shows a non-monotonic variation in ${f}_{avg}\,$at 5 wt.% GLR.

To this point it is clear that at low GLR the cascades self-tune temporally to burst the droplet at increasingly higher ${f}_{avg}\,$when they occur in close vicinity while they tend to suppress ${f}_{avg}\,$when they occur at a distinct location. However, at high GLR, cascades always occur at random locations, which causes the droplet to burst at random frequencies. The global response parameters ${f}_{avg}\,$and ${C}_{max}^{\ast }$ explain the bursting dynamics of a gel droplet as a whole but fail to provide insights on the local dynamics at the rupture location. For instance, it is unclear if the weak region is confined to a particular area or spreads with each cycle. In other words, do ruptures during successive cycles happen exactly at the same location or they are offset? If rupture sites during successive cycles overlapped perfectly, the total cycle time period *T* and ${C}_{max}^{\ast }$ would decay continuously with each cycle. However, this is not the case. For instance, Fig. 6a for 3 wt.% case clearly illustrates that in CAS 1 ${T}_{C5} > {T}_{C4}$, in CAS 2 ${T}_{C4} > {T}_{C3}$ and ${C}_{\max \,,\,c5\,}^{\ast } > \,{C}_{\max \,,\,c2\,}^{\ast }$ and in CAS 3 ${T}_{C6} > {T}_{C1}$. Thus, it is interesting to understand how the weak region develops with each cycle both spatially and temporally as a function of GLR.

### Bursting dynamics at local rupture location and jetting conditions

**Figure 8:**
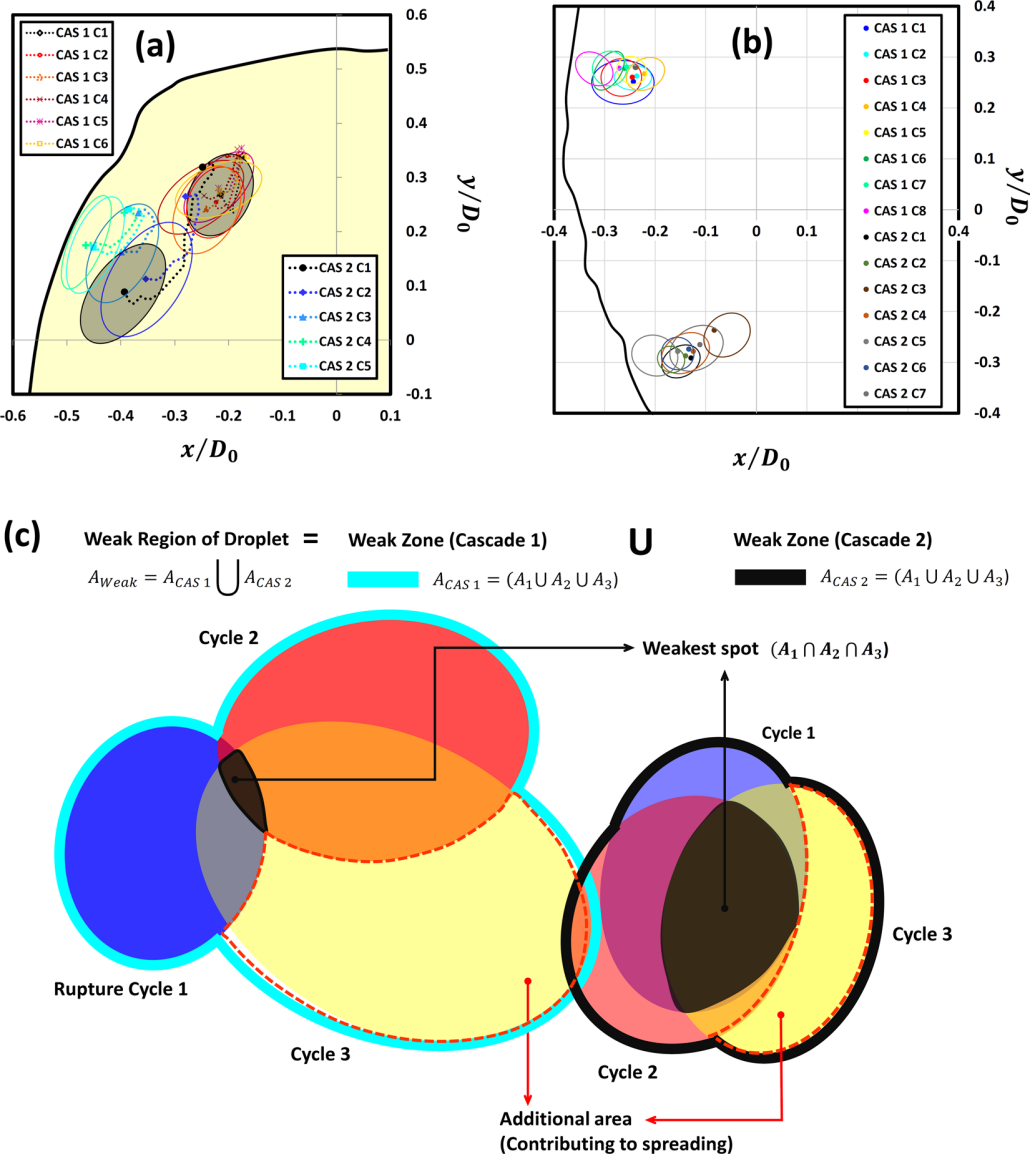
Spatial shifting of rupture sites (with each cycle) during an oscillatory cascade for: (**a**) 3 wt.% gellant loading and (**b**) 5 wt.% gellant loading. (**c**) Schematic showing a set theory based geometrical description of the rupture dynamics (locally at the droplet surface).

Insights on the spatial shifting of rupture sites can be obtained from Fig. 8 which illustrates the sequential growth of weak region with time. Irrespective of the GLR, a key feature of the growth process is that the jet holes during successive cycles do not overlap perfectly but are offset by a certain degree that varies with GLR. For further analysis we assume that the ruptured area ${A}_{n}$ enclosed by the jet hole during ${n}^{th}$ cycle becomes a weak area of droplet. With this assumption, a schematic diagram in Fig. 8c describes the method used for defining the weak region using *set theory*. The weak region comprises of zones that are formed by individual cascades and the growth of a zone after ${n}^{th}$ cycle (in a given cascade) is characterized by three parts: (1) Common intersection of all rupture areas ($\underset{j=1}{\overset{n}{\cap }}{A}_{j}$). As it is common to all cycles, it denotes the weakest spot within the zone that is most vulnerable to undergo rupture in each cycle. In essence this represents akin of an epicenter through which the cascades proceed. A comparison of Fig. 8a and b shows that at 3wt.% the weak spots form in close vicinity such that the weak zones formed by consecutive cascades overlap to a certain extent and weaken a particular area of the droplet (Quad. 2 in Fig. 8a). Contrarily, at 5 wt.% the weak spots are spatially segregated. This reinforces the findings from previous sections that high intensity ruptures at high GLRs (Fig. 4) tend to non-uniformize the shell (Fig. 5 SEM) that randomizes the occurrence of cascades (Quad. 2 and 3 in Fig. 8b). (2) The relative complement of $\underset{j=1}{\overset{n-1}{\cup }}{A}_{j}$ in ${A}_{n}$. This denotes the additional area that is responsible for widening or growth of the weak zone and (3) union of all rupture areas ($\underset{j=1}{\overset{n}{\cup }}{A}_{n}$) that represents the total area of the weak zone formed after $n$ cycles. By combining the aforementioned geometrical parameters the local bursting dynamics of burning ethanol gel droplets can be explained based on the relative spreading factor ${S}^{\ast }(t)$:

1 $${S}^{\ast }(t)=\frac{{A}_{n}{\cup }^{}(\underset{j=1}{\overset{n-1}{\cup }}{A}_{j})\,}{\underset{j=1}{\overset{n-1}{\cup }}{A}_{j}}$$

**Figure 9:**
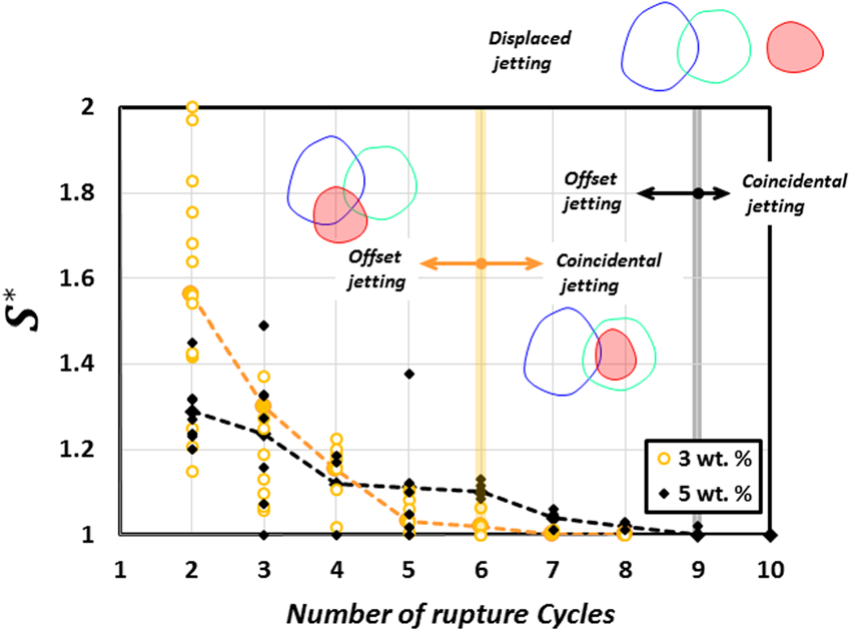
Variation of relative spreading factor ${S}^{\ast }(t)=\frac{{A}_{n}{\cup }^{}(\underset{j=1}{\overset{n-1}{\cup }}{A}_{j})\,}{\underset{j=1}{\overset{n-1}{\cup }}{A}_{j}}$ with cycle count. ${S}^{\ast }(t)$ is averaged over all the cascades occurring during the droplet lifetime. Offset $({S}^{\ast }(t) > 1$), coincidental $({S}^{\ast }(t)=1$) and displaced jetting conditions are shown marked.

Physically, ${S}^{\ast }(t)$ denotes the extent of overlap between the area ruptured at ${n}^{th}\,$cycle and the weak zone formed by previous cycles and predicts the probability of occurrence of jetting at the same location. In particular, ${S}^{\ast }\,$→ 1 signifies that successive ruptures overlap near-perfectly and in the limiting condition of ${S}^{\ast }$ =  1, a jet initiates within the weak zone formed by preceding cycles, thus leading to *coincidental* jetting, while ${S}^{\ast }$ > 1 denotes an *offset jetting* condition. On the other extreme, as spreading factor deviates from unity i.e. ${S}^{\ast }$ ≫ 1 it signifies that the active jetting site has drifted substantially and has high propensity to switch beyond the weak zone ($\underset{j=1}{\overset{n-1}{\cup }}{A}_{j}$) to a nearby location. Subsequently, a limiting cycle with ${A}_{n}{\cap }^{}\,(\underset{j=1}{\overset{n-1}{\cup }}{A}_{j})=0\,\,$then marks an end to a cascade as the jetting now occurs from a completely new location. This appears as *displaced jetting*. Inset in Fig. 9 schematically represents these three jetting conditions i.e. coincidental, offset and displaced jetting. In present experiments, other than displaced jetting, the jet hole after last cycle (end of cascade) is frequently observed to persist for large time scales ~O (500 ms), thus, leading to continuous sustained jetting. This is because as the rupture site is weakened substantially it is unable to recover. A sustained jet hole is shown marked in Fig. 1(l).

Figure 9 shows that irrespective of the GLR the relative spreading factor averaged over all cascades decays monotonically with increasing number of cycles and approaches unity. This suggests that the droplet area influenced by the weak zone becomes progressively weaker with each cycle (due to repeated ruptures). In other words, probability of occurrence of rupture within the weak zone (or coincidental jetting) heightens with each ensuing cycle. As such, once a cascade is initiated it undergoes a gradual transition from offset jetting during initial cycles to coincidental jetting during end cycles. However, the rate decay of ${S}^{\ast }$ varies with the GLR. For instance at 3 wt.% it takes about 5–6 cycles while at 5 wt.% a larger number of ruptures are required (8–9 cycles) before ${S}^{\ast }\,$→ 1. More number of cycles (or ruptures) per cascade at high GLR again points to a stronger shell formed at high GLR.

## Concluding Remarks

Currently, significant effort is directed in understanding the combustion behavior of gel fuel droplets with an aim of enhancing burn rates and energy density of traditional hydrocarbon fuels. This is done either by regulating the ambient conditions or by varying functional properties such as initial droplet diameter, base fuel and gellant type/concentration. However, jetting of fuel vapors is a key phenomenon in combusting gel droplets that has received little attention. In addition to vaporization, jetting enables an advective transport of unreacted fuel vapors from the droplet to the flame. This increases the fuel efflux and shortens F/O mixing timescales, thereby, promoting efficient combustion. In the existing literature^37^, jetting has been reported to occur through isolated or individual events (i.e. a single rupture-recovery) that have significantly short active time periods ${t}_{a}$ ~ *O* (5 ms). However, the novel oscillatory jetting cascades observed in this study persist for a majority of droplet lifetime ~0.1–0.75 ${T}_{0}$ (${T}_{0}$ ~ 3 s: total droplet lifetime). During this time interval the number of cascades vary from ~1–6 while the number of cycles per cascade (*N*) range from ~2–12, with the corresponding total active jetting time period ~*O* (1 s). Thus, given the long durations of active jetting during cascades, they contribute majorly to the efflux of unreacted fuel vapor mass from the droplet domain and hence represent the most critical mode of fuel vapor transport. Previous studies^35, 37–39^ also postulated that jets emanate repeatedly along the same angular direction. In this context, the relative spreading factor ${S}^{\ast }$ introduced in the current study can be used as a quantitative parameter to precisely demarcate the jetting state of a droplet i.e. whether successive jets are off-set, coincidental or completely displaced. Specifically, the limiting case with ${S}^{\ast }$ = 1 provides a necessary condition for recurrent jetting to occur exactly at the same location on the droplet surface.

Key findings indicate that by varying the initial gelling loading rate (GLR) the bursting frequency of the droplet (or jetting frequency) can be altered. At high GLR, a thick-strong-rigid shell is formed where successive cascades occur at randomly dispersed locations on droplet surface and lead to droplet bursting at random frequencies. However, at low GLRs, a thin-weak-flexible shell is formed that is prone to consecutive rupture cascades in same region of the droplet. This leads to droplet bursting at increasingly higher frequencies. Thus, this method of tuning jetting frequency serves a two-fold purpose. First, homogenizing the fuel charge both by altering the droplet trajectory and enhancing the vapor-phase mixing and second, tuning the burn rates through a controlled release of fuel vapors. Since several industrial processes ranging from microfluidics to pharmaceutics to lab-on-a-chip devices utilize gel droplets, our experiments may act as a versatile platform in understanding the fundamental mechanisms in gel based systems.

## Materials and Methods

### Fuel formulation

In present experiments, a non-metallized ethanol gel containing organic gellant is used as the test fuel. Gelled ethanol consists of three parts: (1) Research grade ethanol (99.8% Pure; CAS No. 64-17-5) which is the base fuel, (2) macromolecular Hydroxypropyl Methyl Cellulose (HPMC; CAS No. 9004-65-3) which is the actual organic gellant (OG) and (3) double distilled water that acts as a base solvent for the OG. All chemicals were procured from Sigma-Aldrich Co. Table 1 details the composition of different fuel formulations with gellant loading rate (GLR) varying in a narrow range from 3 wt.% to 6 wt.%. A key consideration while formulating gel propellants is to maximize on the fuel content alongside maintaining a stable gel phase structure. In this regard, for the present three-component system, 3 wt.% OG represents a lower critical concentration while 6 wt.% is the upper critical concentration. A stable ethanol gel was prepared through a three-step process: First, the OG was added to ethanol and mixed thoroughly by hand-stirring for ~2 min. at room temperature. Second, de-ionized water was added to achieve the gel state which was then stirred mechanically using a a three-blade lab impeller at 500 rpm for ~2 min. Finally, the gel was left undisturbed for ~48 hours at room temperature. This rest period is crucial as it facilitates the completion of gel network formation and also enables an examination of the time-dependent stability of gel fuel (i.e. noticeable phase seperation).

### Experimental Methodology

A volume calibrated *µ*-syringe with a 30-gauge ID needle is used to dispense a ∼2.8 µl gel fuel droplet at the end of 80 µm fused quartz fiber. Due to small fiber diameter (less than 100 µm) and low thermal conductivity (1.4 W/m-K at 293 K) of fused quartz any physical or thermal interference due to the suspension fiber can be neglected^45^. Droplet ignition is achieved through a 150 µm Nichrome wire. The heat input required to achieve droplet ignition (i.e. the ignitor operation time) is regulated by a DC electric power source. The combustion behavior of ethanol-gel droplets in pendant mode is investigated at ambient temperature and pressure conditions and under normal gravity.

### Image Acquisition and Post-processing

An ultra-high speed Photron FASTCAM SA-X2 camera attached with a 6.5 X Navitar Zoom lens is used to record the bursting dynamics of an Ethanol/Water/HPMC droplet at 20,000 fps (Exposure time: 60 µs) and at a spatial resolution of 3.8 µm/pixel. Since it is difficult to form spherical droplets with gel fuels, the initial droplet diameter (${D}_{0}$) was calculated as the diameter of a projected area equivalent sphere. In present experiments, ${D}_{0}$ ~ 1.65 ± 0.08 mm with a projection sphericity varying from 0.97 ± 0.02 at 3 wt.% to 0.85 ± 0.06 at 6 wt.%. Projected area was obtained from back-illuminated high speed images using an in-house developed MATLAB code. The error in the accuracy of measurement for mean droplet diameter is within ± 3%. In this work the presented data is averaged over 10 experimental runs.

## Electronic supplementary material

Supplementary Information

Supplementary Video 1

Supplementary Video 2

## References

[1] Maurice, L. Q., Lander, H., Edwards, T. & Harrison III, W. E. Advanced Aviation Fuels: A Look Ahead via a Historical Perspective. *Fuel* 80, 747–756 (2001).

[2] Edwards T (2003). Liquid Fuels and Propellants for Aerospace Propulsion: 19032003. J. Propul. Power.

[3] Natan, B. & Rahimi, S. The status of gel propellants in year 2000. *International Journal of Energetic Materials and Chemical Propulsion* **5**(1–6) (2002).

[4] B. Jr, T. W., Aeroprojects Inc. *Thixotropic liquid propellant compositions with solid storage characteristics*. US Patent 3,470,040 (1969).

[5] Grelecki, C. J., Keeler, R. A. & Szabo Jr., B. US Navy. *Thixotropic gelled bipropellant composition containing sulphated galactose polymer*. US Patent 3,470,042 (1969).

[6] Eric, R. & Daniel, W., Fmc Corp. Pseudo-plastic rocket propellants containing hydrazines with hydroxypropyl cellulose ether. US Patent 3,492,177 (1970).

[7] Allan, B. D. The United States Of America As Represented By The Secretary Of The Army. *Gelled monomethylhydrazine thixotropic fuel*. US Patent 4,039,360 (1977).

[8] Stanley, T., Thiokol Corp. Heterogeneous monopropellant compositions and thrust producing method. US Patent 3,921,394 (1975).

[9] Fox, R., Lambert, G. & Rains, W., US Air Force. *Rocket propellant and method*. US Patent 3,717,518 (1973).

[10] Allan, B. D., Hubbuch, T. N. & Walter, W. Gelled monopropellant containing hydrazine and a non-hypergolic acid gas gelling agent. US Patent 3,551,226 A (1970).

[11] Hodge, K., Crofoot, T. & Nelson, S. Gelled propellants for tactical missile applications. 35^th^ Joint Propulsion Conference and Exhibit, Los Angeles, CA, USA *AIAA paper* 1999–2976 (1999).

[12] Rahimi S, Natan B (2000). Flow of gel fuels in tapered injectors. Journal of propulsion and power.

[13] Rahimi, S. & Natan, B. Atomization characteristics of gel fuels. *AIAA paper* 98–3830 (1998).

[14] Rahimi S, Natan B (2000). Numerical Solution of the Flow of Power‐Law Gel Propellants in Converging Injectors. Propellants, Explosives, Pyrotechnics.

[15] Chojnacki, K. T. & Feikema, D. A. Atomization studies of gelled bipropellant simulants using planar laser induced fluorescence. In *AIAA, ASME, SAE, and ASEE, Joint Propulsion Conference and Exhibit, San Diego, CA, USA* 1995–2423 (1995).

[16] Green, J. M., Rapp, D. C. & Roncace, J. *Flow visualization of a rocket injector spray using gelled propellant simulants*. Sverdrup Technology Incorporated, Lewis Research Center Group Report. NASA-CR-187142, E-6276, NAS 1.26:187142, AIAA PAPER 91–2198 (1991).

[17] Urbon, B. C. *Atomization and combustion of a gelled, metallized slurry fuel*. Doctoral dissertation, Monterey, California. Naval Postgraduate School (1992).

[18] Solomon Y, Natan B (2006). Experimental investigation of the combustion of organic-gellant-based gel fuel droplets. Combustion Science and Technology.

[19] Solomon Y, Natan B, Cohen Y (2009). Combustion of gel fuels based on organic gellants. Combustion and Flame.

[20] Law CK (1978). Internal boiling and superheating in vaporizing multicomponent droplets. AIChE J..

[21] Wang CH, Law CK (1985). Microexplosion of fuel droplets under high pressure. Comb. Flame.

[22] Wang CH, Liu HQ, Law CK (1984). Combustion and microexplosion of freely falling multicomponent droplets. Comb. Flame.

[23] Miglani A, Basu S, Kumar R (2014). Insight into instabilities in burning droplets. Physics of Fluids (1994-present).

[24] Miglani A, Basu S, Kumar R (2014). Suppression of instabilities in burning droplets using preferential acoustic perturbations. Comb. Flame.

[25] Miglani A, Basu S (2015). Effect of particle concentration on shape deformation and secondary atomization characteristics of a burning nanotitania dispersion droplet. J. Heat Transfer.

[26] Miglani A, Basu S (2015). Coupled mechanisms of precipitation and atomization in burning nanofluid fuel droplets. Scientific reports.

[27] Basu S, Miglani A (2016). Combustion and heat transfer characteristics of nanofluid fuel droplets: A short review. International Journal of Heat and Mass Transfer.

[28] Shinjo J, Xia J, Ganippa LC, Megaritis A (2014). Physics of puffing and microexplosion of emulsion fuel droplets. Physics of Fluids.

[29] Rao, D., Karmakar, S. & Som, S. K. Puffing and micro-explosion behavior in combustion of butanol/Jet A-1 and acetone-butanol-ethanol (ABE)/Jet A-1 fuel droplets. *arXiv preprint arXiv:1611.10199* (2016).

[30] Avulapati MM, Ganippa LC, Xia J, Megaritis A (2016). Puffing and micro-explosion of diesel–biodiesel–ethanol blends. Fuel.

[31] He B, Nie W, He H (2012). Unsteady combustion model of non-metalized organic gel fuel droplet. Energy & Fuels.

[32] Solomon Y, DeFini SJ, Pourpoint TL, Anderson WE (2012). Gelled monomethyl hydrazine hypergolic droplet investigation. Journal of Propulsion and Power.

[33] Liu Z, Hu X, He Z, Wu J (2012). Experimental study on the combustion and microexplosion of freely falling gelled unsymmetrical dimethylhydrazine (UDMH) fuel droplets. Energies.

[34] Arnold, R. & Anderson, W. E. Droplet burning of JP-8/silica gels. In *48th AIAA Aerospace Sciences Meeting Including the New Horizons Forum and Aerospace Exposition*, 4–7 Jan, Orlando, Florida, USA AIAA paper 2010–421 (2010).

[35] Kunin A, Natan B, Greenberg JB (2010). Theoretical model of the transient combustion of organic-gellant-based gel fuel droplets. Journal of Propulsion and Power.

[36] Nachmoni GAD, Natan B (2000). Combustion characteristics of gel fuels. Combustion Science and Technology.

[37] Cho KY, Pourpoint TL, Son SF, Lucht RP (2013). Microexplosion Investigation of Monomethylhydrazine Gelled Droplet with OH Planar Laser-Induced Fluorescence. Journal of Propulsion and Power.

[38] Mishra DP, Patyal A (2012). Effects of initial droplet diameter and pressure on burning of ATF gel propellant droplets. Fuel.

[39] Mishra DP, Patyal A, Padhwal M (2011). Effects of gellant concentration on the burning and flame structure of organic gel propellant droplets. Fuel.

[40] Karagozian AR (2016). Acoustically Coupled Combustion of Liquid Fuel Droplets. Applied Mechanics Reviews.

[41] Sevilla-Esparza CI (2014). Droplet combustion in the presence of acoustic excitation. Combustion and Flame.

[42] Dattarajan S, Lutomirski A, Lobbia R, Smith OI, Karagozian AR (2006). Acoustic excitation of droplet combustion in microgravity and normal gravity. Combustion and Flame.

[43] Valentini, D. *et al*. Partial Extinction and the Rayleigh Index in Acoustically Driven Fuel Droplet Combustion. In *APS Meeting Abstracts* (2014).

[44] Blaszczyk J (1991). Acoustically disturbed fuel droplet combustion. Fuel.

[45] Law, C. K. *Combustion physics*. Cambridge University Press (2010).

