# Supplementary material for: Oscillatory bursting of gel fuel droplets in a reacting environment
Source: Sci Rep. 2017 Jun 12;7:3088. doi: 10.1038/s41598-017-03221-x (PMC5468320; doi:10.1038/s41598-017-03221-x)
Supplement: Supplementary file 1 — Supplementary Information [file 41598_2017_3221_MOESM1_ESM.pdf]

SUPPLEMENTARY MATERIAL

Oscillatory bursting of gel fuel droplets in a reacting environment

Ankur Miglani, Purushothaman Nandagopalan, Jerin John and Seung Wook Baek*

Department of Aerospace Engineering, School of Mechanical and Aerospace Engineering, Korea Advanced Institute of Science and Technology (KAIST), Daejeon, Republic of Korea – 34141

Oscillatory flame Response:

Typical flame envelope disruptions resulting from oscillatory bursting are shown in Supplementary Video 2 and expanded below in the Fig. 1. As reported in Ref. [33], depending on the jetting intensity (i.e. maximum jet speed $U = {}^{\Delta r}/_{\Delta t}$) a jet may either distort the flame envelope ($U \sim$ 0.34 m/s) or break the flame envelope (local extinction; $U \sim$ 0.48 m/s) or form a fireball outside the flame envelope. Here, Δr represents the *displacement* of the flame front during the active jetting period in the direction of jet (as jet hits the flame front). This is marked in Fig. 1 below, which also shows how the jet speed is estimated. Fig. 1 illustrates how the flame front undergoes distortion continuously as it is subjected to different jetting cycles in an oscillatory cascade. The entry of fuel jet into the gaseous phase is apparent at the onset of each cycle. Subsequently, as jet hits the flame front, cycles 1 and 2 with $U \sim$ 0.22 m/s and 0.29 m/s respectively tend to corrugate the flame front only slightly while cycles 3, 4, 5 and 6 with $U \sim$ 1.3 m/s and 0.98 m/s 0.76 m/s and 0.82 m/s respectively induce severe distortion of the flame envelope. The extent of distortion in the flame front resulting from jetting events is quantified by the dimensionless parameter $\beta = {}^{R_{f,max}}/_{R_f}$. β essentially represents the normalized flame stand-off distance where $R_{f,max}$ is taken as the maximum radial distance from the droplet centroid to the flame edge measured along the vertical axis, while R_f is its magnitude in the quasi-steady flame state prior to onset of jetting events; as marked in Fig. 1 below.

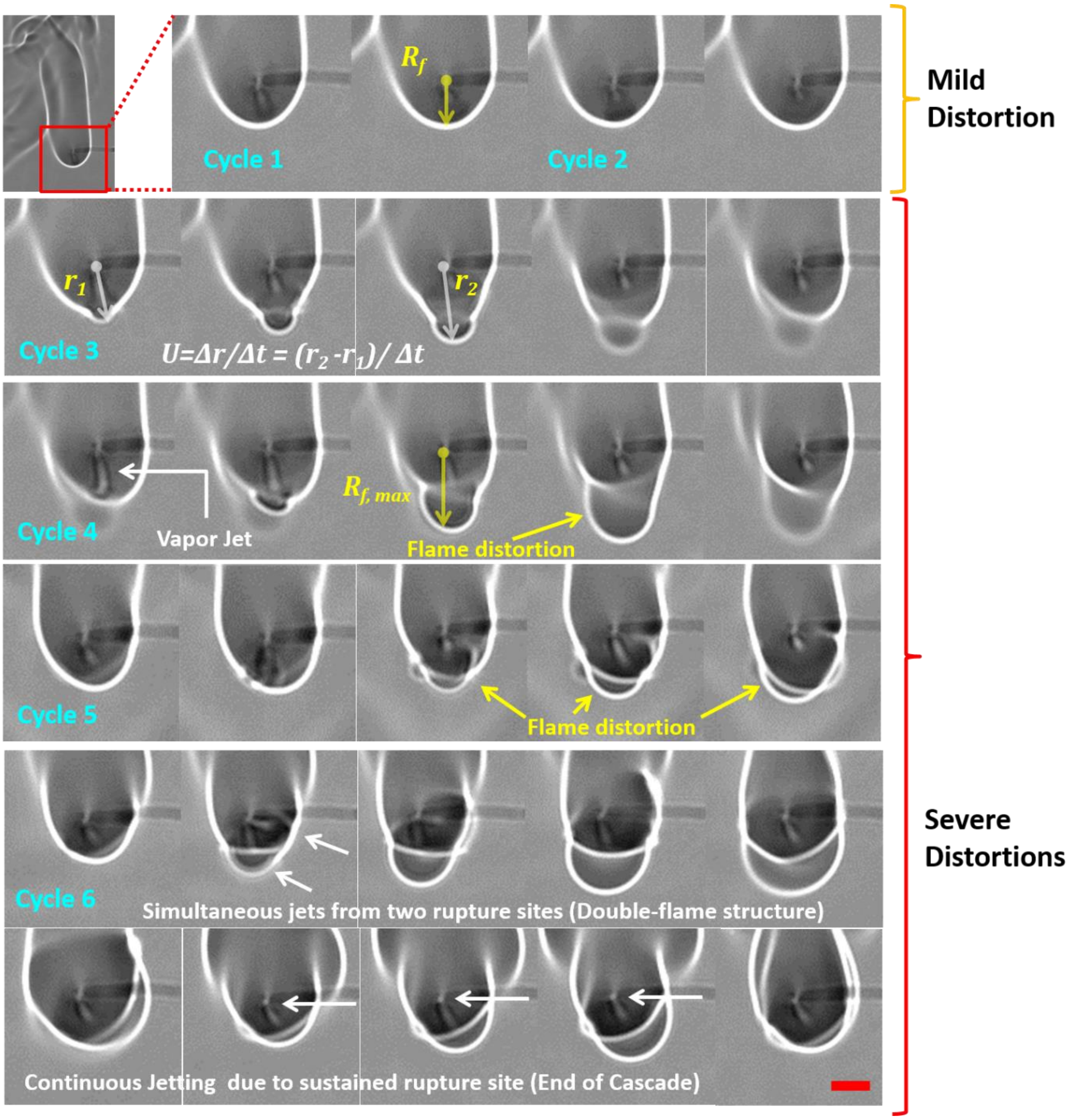

Fig. 1. Dynamic response of envelope diffusion flame (for a 3 wt.% ethanol gel droplet) during an oscillatory rupture cascade with 6 jetting cycles. This shows that cascade constitutes a series of aperiodically occurring jetting events that lead to both flame envelope distortion and possible localized extinction depending on the jetting intensity (jet speed $U{=}\Delta r \Delta t/$). For the image sequence (from top to bottom and left to right) the images are time instants: Cycle 1: t = 0ms, 1ms. Cycle2: 13, 14 ms. Cycle 3: 51, 53, 58, 78, 93 ms. Cycle 4: 94, 95, 98, 108, 128 ms. Cycle 5: 186, 189, 192, 196, 203 ms. Cycle 6: 288, 294, 299, 308, 328, 358, 388, 398, 408, 438 ms. The scale bar equals 5 mm.

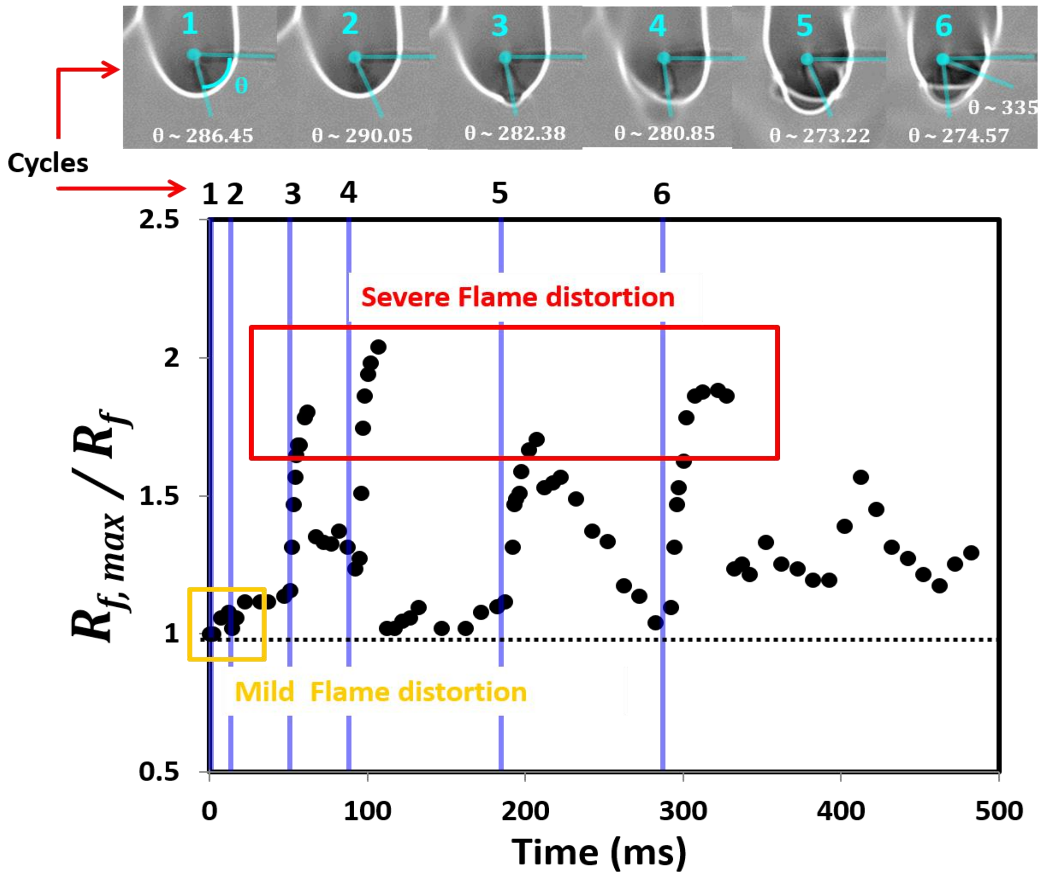

Fig. 2. Temporal variation in normalized flame stand-off distance $Rf{,}x$ / Rf during an oscillatory cascade with 6 jetting cycles. Physically, the distorted flame structure can be characterized as mild distorted or severely distorted based on the magnitude of $Rf{,}x$ / Rf.

Fig. 2 indicates that despite irregular jetting disruptions, the overall oscillatory flame response resulting from a cascade is clearly reflected by harmonic oscillations in β. Furthermore, the fact that rupture holes during successive cycles occur in the same region and overlap to a large extent is also evident at the flame scale, since jets during all cycles are observed to emanate along the same angular direction. There is negligible variation (within $\sim$13°) in the angular position of jets.

Shell bursting Dynamics:

Stages exhibiting a sequential transition from bubble growth/formation to shell rupture and jetting is shown schematically in Fig. 3 and the critical length scales used in the analysis are shown marked.

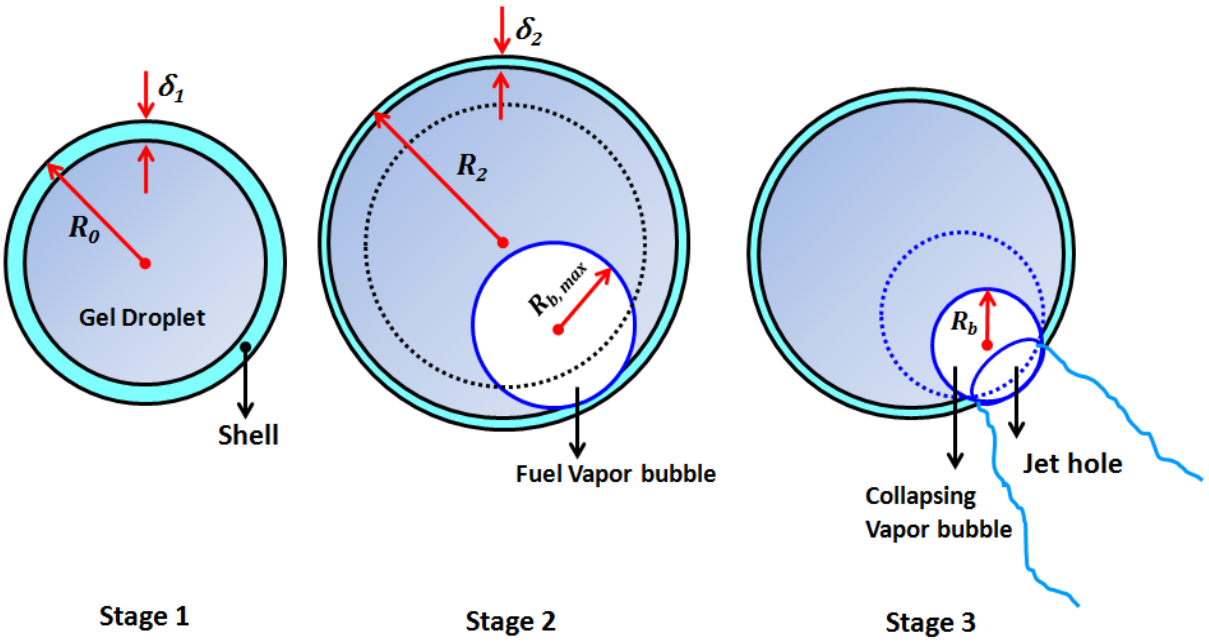

Fig. 3. Schematic showing stages during the combustion of an organic-gellant based ethanol fuel droplet. Stage 1: Shell formation, Stage 2: Internal boiling and droplet swelling alongside thinning of shell and Stage 3: Shell rupture and jetting. Geometric parameters are shown marked. R_0 or R_1 is the initial droplet radius.

The droplet volume (V_D) before and after the bubble formation is given by:

Before bubble formation (Stage 1): $V_{D1} = V_{f1} + V_{g1}$ (1)

After bubble formation (Stage 2) : $V_{D2} = V_{bubble} + \left(V_{f1} - V_{f,\ lg}\right) + V_{g2}$ (2)

Here, V_f, V_g and $V_{bubble}$are respectively the volumes of liquid fuel, organic gellant and the bubble while$V_{f,\ lg}$ is the quantity of liquid fuel that gasified to form the bubble. During the swelling period the evaporative mass flux at the droplet surface is zero. Therefore, fuel mass conservation at anytime instant during the bubble growth period can be expressed as:

$$m_{bubble} = \rho_v V_{bubble} = \rho_f V_{f,\ lg} \quad (3)$$

Further, as the organic gellant is non-volatile the gellant volume remains constant ($V_{g1} = V_{g2}$). From equations (1), (2) and (3) the change in droplet volume ΔV is then given by:

$$\Delta V\ (V_{D2} - V_{D1}) = V_{bubble}(1 - \frac{\rho_v}{\rho_f}) \quad (4)$$

Assuming that the droplet temperature is uniform and constant and equal to the boiling point of high volatility species (ethanol: T=78.5°C at 0.1 MPa), the density ratio $\frac{\rho_v}{\rho_f} \ll 1$ ($\rho_v \approx 1.6$ Kg/m^3 and $\rho_f \approx 725$ Kg/m^3). Thus, the change in internal liquid volume is negligible compared to the change in vapour volume or in other words the change in droplet volume is essentially due to bubble formation$\Delta V_D \approx V_{bubble}$. Based on this analysis, as the droplet swells the gellant mass conservation demands that:

$$\delta_2 = \left(\frac{R_1}{R_2}\right)^2 \delta_1 \tag{5}$$

where, R_1 and δ_1 are respectively the droplet radius and shell thickness initially (prior to bubble formation) while R_2 and δ_2 are the corresponding values after swelling.

Next, as the droplet expands, the gellant shell thins downs further and ruptures when the tensile stress applied to the spherical shell reaches the rupture stress (~yield point of the material). The critical hoop (or circumferential) stress at rupture (Stage 3: Fig. 3) is given by:

$$\tau = \frac{\Delta P.\ R}{2\,\delta} \tag{6}$$

From the experimental data, droplet radius at first rupture is $R \sim O$ (1mm). As reported by He et. al [28] the initial shell thickness for millimeter sized droplets is $\delta_0 \sim O$ (5 µm). Given that at rupture $R/_{R_0} \sim 1.25$ and using Eq. (5) the shell thickness at rupture is of the order of $\delta \sim O$ (3 µm). Assuming a quasi-steady balance between the pressure difference across the shell and tension in the shell at rupture ΔP can be estimated using the expression:

$$\Delta P = P_v - P_0 = {}^{\rho_v U^2}/_2 \tag{7}$$

Using the representative values from present experiments and as reported by Cho et. al [33] the jet speed following rupture $U \sim O$ (1 m/s), vapour density $\rho_v \sim 1.6$ Kg/m^3 . The pressure difference across shell is calculated to be $\Delta P \sim$ O (1 Pa). Through this order of magnitude analysis and using Eq. (6) the tensile stress at rupture is $\tau \sim O$ (1 kPa). This is very close to the magnitude of 2 kPa as reported by He et. al [28].

Following rupture, the fuel mass conservation demands that rate of bubble collapse rate equals the mass efflux of fuel vapors during the jetting period (t_a):

$$-\dot{m}_b (= -\rho_v \frac{dV_b(t)}{dt}) = \rho_v . A(t). U(t) \text{ or}$$

$$-\int R_b^2 \, dR = \frac{1}{4\pi} \int A(t). U(t) dt \tag{8}$$

where, $A(t), U(t)$ and $R_b(t)$ are the temporally varying area of the jet hole, the jet speed and the bubble radius respectively. Fig. 1 of the manuscript shows that in a single jetting cycle the jet hole area expands and then contracts till its complete recovery. Consequently,

this would cause a variation in fuel mass flux. However, for a first order analysis we assume that the jet hole area and the jet speed remain constant during the active jetting period. Using Eq. (7) $U = \sqrt{2(P_v - P_0)/\rho_v}$ and given the initial condition that at rupture onset (time $t = 0$) the bubble is at its maximum radius $R_{b,max}$, eq. (8) above can be used to estimate the variation in bubble radius during the jetting time period:

$$R_b\ (t) = \left(R_{b,max}^3 - \frac{3}{4\pi}\sqrt{\frac{2(P_v - P_0)}{\rho_v}}\ A.t\right)^{\frac{1}{3}} \tag{9}$$

Finally, note that the temporal variation of bubble radius is intrinsically a function of rheophysical properties of the shell as the pressure difference is related to shell thickness and tensile stress at rupture through eq. (6).

References:

[28] He, B., Nie, W. and He, H. Unsteady combustion model of non-metalized organic gel fuel droplet. *Energy & Fuels*, 26(11), 6627-6639 (2012).

[33] Cho, K.Y., Pourpoint, T.L., Son, S.F. and Lucht, R.P. Microexplosion Investigation of Monomethylhydrazine Gelled Droplet with OH Planar Laser-Induced Fluorescence. *Journal of Propulsion and Power* 29(6), pp.1303-1310 (2013).

[34] Mishra, D.P. and Patyal, A., Effects of initial droplet diameter and pressure on burning of ATF gel propellant droplets. *Fuel* 95, 226-233 (2012).

[35] Mishra, D.P., Patyal, A. and Padhwal, M. Effects of gellant concentration on the burning and flame structure of organic gel propellant droplets. *Fuel 90*(5), 1805-1810 (2011).

Video 1: Aperiodic bursting of a combusting 3 wt. % ethanol-gel droplet undergoing an oscillatory rupture cascade.

Video 2: Oscillatory response of envelope diffusion flame resulting from an oscillatory bursting cascade in a burning 3wt. % ethanol gel droplet.
